# Multiple Long-Read Sequencing Survey of Herpes Simplex Virus Dynamic Transcriptome

**DOI:** 10.3389/fgene.2019.00834

**Published:** 2019-09-24

**Authors:** Dóra Tombácz, Norbert Moldován, Zsolt Balázs, Gábor Gulyás, Zsolt Csabai, Miklós Boldogkői, Michael Snyder, Zsolt Boldogkői

**Affiliations:** ^1^Department of Medical Biology, Faculty of Medicine, University of Szeged, Szeged, Hungary; ^2^Department of Genetics, School of Medicine, Stanford University, Stanford, CA, United States

**Keywords:** herpesviruses, herpes simplex virus, long-read sequencing, direct RNA sequencing, Pacific Biosciences, Oxford Nanopore Technologies, transcript isoforms

## Abstract

Long-read sequencing (LRS) has become increasingly important in RNA research due to its strength in resolving complex transcriptomic architectures. In this regard, currently two LRS platforms have demonstrated adequate performance: the Single Molecule Real-Time Sequencing by Pacific Biosciences (PacBio) and the nanopore sequencing by Oxford Nanopore Technologies (ONT). Even though these techniques produce lower coverage and are more error prone than short-read sequencing, they continue to be more successful in identifying polycistronic RNAs, transcript isoforms including splice and transcript end variants, as well as transcript overlaps. Recent reports have successfully applied LRS for the investigation of the transcriptome of viruses belonging to various families. These studies have substantially increased the number of previously known viral RNA molecules. In this work, we used the Sequel and MinION technique from PacBio and ONT, respectively, to characterize the lytic transcriptome of the herpes simplex virus type 1 (HSV-1). In most samples, we analyzed the poly(A) fraction of the transcriptome, but we also performed random oligonucleotide-based sequencing. Besides cDNA sequencing, we also carried out native RNA sequencing. Our investigations identified more than 2,300 previously undetected transcripts, including coding, and non-coding RNAs, multi-splice transcripts, as well as polycistronic and complex transcripts. Furthermore, we found previously unsubstantiated transcriptional start sites, polyadenylation sites, and splice sites. A large number of novel transcriptional overlaps were also detected. Random-primed sequencing revealed that each convergent gene pair produces non-polyadenylated read-through RNAs overlapping the partner genes. Furthermore, we identified novel replication-associated transcripts overlapping the HSV-1 replication origins, and novel LAT variants with very long 5’ regions, which are co-terminal with the LAT-0.7kb transcript. Overall, our results demonstrated that the HSV-1 transcripts form an extremely complex pattern of overlaps, and that entire viral genome is transcriptionally active. In most viral genes, if not in all, both DNA strands are expressed.

## Introduction

Next-generation short-read sequencing (SRS) technology has revolutionized the research fields of genomics and transcriptomics due to its capacity of sequencing a large number of nucleic acid fragments simultaneously at a relatively low cost (Mortazavi et al., 2008; Wang et al., 2009; Djebali et al., 2012). However, SRS technologies have inherent limitations both in genome and transcriptome analyses. This approach does not perform adequately in mapping repetitive elements and GC-rich DNA sequences, or in discriminating paralogous sequences. In transcriptome research, SRS techniques have difficulties in identifying multi-spliced transcripts, overlapping transcripts, transcription start site (TSS), and transcription end site (TES) isoforms, as well as multigenic RNA molecules.

Long-read sequencing (LRS) techniques can resolve these obstacles. The LRS technology is able to read full-length RNA molecules, therefore it is ideal for application in the analysis of complex transcriptomic profiles. Currently two techniques are available in the market, the California-based Pacific Biosciences (PacBio) and the British Oxford Nanopore Technologies (ONT) platforms. The PacBio approach is based on single-molecule real-time (SMRT) technology, while the ONT platform utilizes the nanopore sequencing concept. Both techniques have already been applied for the structural and dynamic transcriptomic analysis of various organisms (Byrne et al., 2017; Chen et al., 2017; Cheng et al., 2017; Li et al., 2018; Nudelman et al., 2018; Wen et al., 2018; Zhang et al., 2018; Jiang et al., 2019; Zhao et al., 2019), including viruses (Boldogkői et al., 2019b), such as herpesviruses (Tombácz et al., 2015; O’Grady et al., 2016; Tombácz et al., 2016; Balázs et al., 2017a; Balázs et al., 2017b; Moldován et al., 2017b; Tombácz et al., 2017b; Tombácz et al., 2017a; Tombácz et al., 2018b; Depledge et al., 2019), poxviruses (Tombácz et al., 2018a), baculoviruses (Moldován et al., 2018b), retroviruses (Moldován et al., 2018a), coronaviruses (Viehweger et al., 2019), and circoviruses (Moldován et al., 2017a). Additionally, the ONT technology is capable of sequencing DNA and RNA in its native form, allowing epigenetic and epitranscriptomic analysis (Wongsurawat et al., 2018; Liu et al., 2019; Shah et al., 2019).

Herpes simplex virus type 1 (HSV-1) is a human pathogenic virus belonging to the *Alphaherpesvirinae* subfamily of the *Herpesviridae* family. Its closest relatives are the HSV-2, the Varicella-zoster virus (VZV), and the animal pathogen pseudorabies virus (PRV). The most common symptom of HSV-1 infection is cold sores, which can recur from latency causing blisters primarily on the lips. HSV-1 may cause acute encephalitis in immunocompromised patients. The ability of herpesviruses to establish lifelong latency within the host organism significantly contributes to their evolutionary success: according to WHO’s estimates, more than 3.7 billion people under the age of 50 are infected with HSV-1 worldwide (Looker et al., 2015).

HSV-1 has a 152-kbp linear double-stranded DNA genome that is composed of unique and repeat regions. Both the long (UL) and the short (US) unique regions are flanked by inverted repeats (IRLs and IRSs, respectively) (Macdonald et al., 2012). The viral genome is transcribed by the host RNA polymerase in a cascade-like manner producing three kinetic classes of transcripts and proteins: immediate-early (IE), early (E), and late (L) (Harkness et al., 2014). IE genes encode transcription factors required for the expression of E and L genes. E genes mainly code for proteins playing a role in DNA synthesis, whereas L genes specify structural elements of the virus. Earlier studies and *in silico* annotations have revealed 89 mRNAs, 10 non-coding (nc)RNAs (Rajčáni et al., 2004; McGeoch et al., 2006; Macdonald et al., 2012; Lim, 2013; Hu et al., 2016), and 18 microRNAs (Du et al., 2015). Our recent study (Tombácz et al., 2017b) based on PacBio RS II sequencing has identified additional 142 transcripts and transcript isoforms, including ncRNAs. The detection and the kinetic characterization of HSV-1 transcriptome face an important challenge because of the overlapping and polycistronic nature of the viral transcripts. Polycistronic transcription units are different from those of bacterial operons, in that the downstream genes on multigenic transcripts are untranslated because herpesvirus mRNAs use cap-dependent translation initiation (Merrick, 2004). The majority of herpesvirus transcripts are organized into tandem gene clusters generating overlapping transcripts with co-terminal TESs. The *ul41-44* genomic region of HSV-1 does not follow this rule, since these genes are primarily expressed as monocistronic RNA molecules. Our earlier study has revealed that these genes also produce low-abundance bi- and polycistronic transcripts. Alternatively, many HSV-1 genes, which were believed to be exclusively expressed as parts of multigenic RNAs, have also been shown to specify low-abundance monocistronic transcripts (Tombácz et al., 2017b).

SRS technologies have become useful tools for the analysis of transcriptomes. However, conventionally applied SRS platforms cannot reliably distinguish between multi-spliced transcript isoforms, and TSS variants, as well as between embedded transcripts and their host RNAs, etc. Additionally, SRS, even if applied in conjunction with auxiliary techniques such as RACE analysis, has limitations in detecting multigenic transcripts, including polycistronic RNAs and complex transcripts (cxRNAs; containing genes standing in opposite orientations). LRS is able to circumvent these problems. Both PacBio and ONT approaches are capable of reading cDNAs generated from full-length transcripts in a single sequencing run and permit mapping of TSSs and TESs with base-pair precision. The most important disadvantage of LRS compared to SRS techniques is lower coverage. In PacBio sequencing, if any errors occur in raw reads, they are easily corrected thanks to the very high consensus accuracy of this technique (Miyamoto et al., 2014). Thus, it is only a widespread myth that SMRT sequencing is too error prone to be used for precise sequence analysis. The precision of basecalling is substantially lower for ONT platform than that of PacBio, but the former technique is far more cost-effective, and yields both higher throughput and longer reads. The high error rate of the ONT technique can be circumvented by obtaining high sequence coverage. Nonetheless, this latter problem is not critical in transcriptome research if the genome sequence of the examined organism has already been annotated.

A diverse collection of methods and approaches have already been employed for the investigation of herpesvirus transcriptomes, including *in silico* detection of open reading frames (ORFs) and cis-regulatory motifs, Northern-blot analysis (Costa et al., 1984; Sedlackova et al., 2008), S1 nuclease mapping (McKnight, 1980; Rixon and Clements, 1982), primer extension (Perng et al., 2002; Naito et al., 2005), real-time reverse transcription-PCR (RT^2^-PCR) analysis (Tombácz et al., 2009), microarrays (Stingley et al., 2000), Illumina sequencing (Harkness et al., 2014; Oláh et al., 2015), PacBio RS II (O’Grady et al., 2016; Tombácz et al., 2017b), and Sequel sequencing, as well as ONT MinION cDNA and direct RNA sequencing (Boldogkői et al., 2018; Prazsák et al., 2018; Depledge et al., 2019).

In this study, we report the application of PacBio Sequel and ONT MinION long-read sequencing technologies for the characterization of the HSV-1 lytic transcriptome. We used an amplified isoform sequencing (Iso-Seq) protocol of PacBio that was based on PCR amplification of cDNAs prior to sequencing. We used both cDNA and direct (d)RNA sequencing on the ONT platform. Additionally, we applied Cap-selection for ONT sequencing. In order to identify non-polyadenylated transcripts, we also applied random oligonucleotide primer-based RT in addition to the oligo(dT)-priming. Furthermore, the latter technique is more efficient for the mapping of the TSSs, and it is useful for the validation of novel RNA molecules. Our intentions of using novel LRS techniques were to analyze the dynamic viral transcriptome, to generate a higher number of sequencing reads, and to identify novel transcripts that had been undetected in our earlier PacBio RS II-based approach. Furthermore, in this report, we also reanalyzed our earlier results that were obtained using a single-platform method (Tombácz et al., 2017b).

## Materials and Methods

### Cells and Viral Infection

The strain KOS of HSV-1 was propagated on an immortalized kidney epithelial cell line (Vero) isolated from the African green monkey (*Chlorocebus sabaeus*). Vero cells were cultivated in Dulbecco’s modified Eagle medium supplemented with 10% fetal bovine serum (Gibco Invitrogen) and 100 μl penicillin–streptomycin 10K/10K mixture (Lonza)/ml and 5% CO_2_ at 37°C. The viral stocks were prepared by infecting rapidly-growing semi-confluent Vero cells at a multiplicity of infection (MOI) of 1 plaque-forming unit (pfu)/cell, followed by incubation until a complete cytopathic effect was observed. The infected cells were then frozen and thawed three times. The cells were then centrifuged at 10,000 ×g for 15 min using low-speed centrifugation. For the sequencing studies, cells were infected with MOI = 1, incubated for 1 h. This was followed by removal of the virus suspension and a PBS washing step. Next, the cells were supplied with a fresh culture medium and were then incubated for 1, 2, 4, 6, 8, 10, 12, or 24 h.

### RNA Isolation

The total RNA samples were purified from cells using the NucleoSpin^®^ RNA kit (**Table 1**) according to the kit’s manual and our previously described methods (Boldogkői et al., 2018). The RNA samples were quantified using the Qubit^®^ 2.0 Fluorometer and were stored at -80°C until use. The samples taken from each experiment were then mixed for sequencing. Samples were subjected to ribodepletion for the random primed sequencing, while selection of the poly(A)^+^ RNA fraction was being carried out for polyA-sequencing. All experiments were performed in accordance with the relevant guidelines and regulations.

**Table 1:** Summary of the kits used for RNA preparation and quantitation.

Method		Kit	Company
RNA purification	Total RNA extraction	NucleoSpin RNA	Macherey Nagel
	PolyA(+) RNA isolation	Oligotex mRNA Mini Kit	Qiagen
	Ribodepletion	Ribo-ZeroTM Magnetic Kit H/M/R	Epicentre/Illumina
Concentration measurement	Total RNA	Qubit RNA BR Assay Kit	Life Technologies
	PolyA(+) RNA	Qubit RNA HS Assay Kit	
	rRNA depleted RNA		
Elimination of non-capped RNAs	5’-phasopahte-dependent-exonuclease digestion	Terminator™ 5′-Phosphate-Dependent Exonuclease	Epicentre/Lucigen

### Pacific Biosciences RS II and Sequel Platforms—Sequencing of the Polyadenylated RNA Fraction or the Total Transcriptome

The Clontech SMARTer PCR cDNA Synthesis Kit was used for cDNA preparation according to the PacBio Isoform Sequencing (Iso-Seq) protocol. For the analysis of relatively short viral RNAs, the ‘No-size selection’ method was used and samples were run on the RSII and Sequel platforms, both. The SageELF™ and BluePippin™ Size-Selection Systems (Sage Science) were also used to carry out size-selection for capturing the potential long, rare transcripts. The reverse transcription (RT) reactions were primed by using the oligo(dT) from the SMARTer Kit. However, we also used random primers for a non-size selected sample to detect non-polyadenylated RNAs. The cDNAs were amplified by the KAPA HiFi Enzyme from KAPA Biosystems, according to PacBio’s recommendations (Balázs et al., 2017b; Tombácz et al., 2018b). The SMRTbell libraries were generated using PacBio Template Prep Kit 1.0. For binding the DNA polymerase and annealing the sequencing primers, the DNA/Polymerase Binding Kit P6-C4 and v2 primers, as well as the Sequel Sequencing Kit and v3 primers were used for the RSII and Sequel sequencing, respectively. The DNA/Polymerase Binding Kit P6-C4 and v2 primers were used for binding DNA polymerase and for annealing sequencing primers. Whereas, the Sequel Sequencing kit and v3 primers were used for RSII and Sequel sequencing.

The polymerase-template complexes were bound to MagBeads with the PacBio MagBead Binding Kit. Samples were loaded onto the RSII SMRT Cell 8Pac v3 or Sequel SMRT Cell 1M. The movie time was 240 or 360 min *per* SMRT Cell for the RSII, while 600-min movie time was set to the Sequel run.

### Oxford Nanopore Minion Platform—cDNA Sequencing Using Oligo(dT) or Random Primers

#### Regular (No Cap Selection) Protocol

The 1D Strand switching cDNA by ligation protocol (Version: SSE_9011_v108_revS_18Oct2016) from the ONT was used for sequencing HSV-1 cDNAs on the MinION platform. The ONT Ligation Sequencing Kit 1D (SQK-LSK108) was applied for the library preparation using the recommended oligo(dT) primers, or custom-made random oligonucleotides, as well as the SuperScipt IV enzyme for the RTs. The cDNA samples were subjected to PCR reactions with KAPA HiFi DNA Polymerase (Kapa Biosystems) and Ligation Sequencing Kit Primer Mix (part of the 1D Kit). The NEBNext End repair/dA-tailing Module (New England Biolabs) was used for the end repair, whereas the NEB Blunt/TA Ligase Master Mix (New England Biolabs) was utilized for the adapter ligation. The enzymatic steps (e.g.: RT, PCR, and ligation) were carried out in a Veriti Cycler (Applied Biosystems) according to the 1D protocol (Moldován et al., 2018b; Tombácz et al., 2018b). The Agencourt AMPureXP system (Beckman Coulter) was used for the purification of samples after each enzymatic reaction. The quantity of the libraries was checked using the Qubit Fluorometer 2.0 and the Qubit (ds)DNAHS Assay Kit (Life Technologies). The samples were run on R9.4 SpotON Flow Cells from ONT.

#### Cap Selection Protocol

The TeloPrime Full-Length cDNA Amplification Kit (Lexogen) was used for generating cDNAs from 5’ capped polyA^(+)^ RNAs. RT reactions were carried out with oligo(dT) primers (from the kit) or random hexamers (custom made) using the enzyme from the kit. A specific adapter (capturing the 5’ cap structure) was ligated to cDNAs (25°C, overnight), then the samples were amplified by PCR using the Enzyme Mix and the Second-Strand Mix from the TeloPrime Kit. The reactions were performed in a Veriti Cycler and the samples were purified on silica membranes (TeloPrime Kit) after the enzymatic reactions. The Qubit 2.0 and the Qubit dsDNA HS quantitation assays (Life Technologies) were used for measuring the concentration of the samples. A quantitative PCR reaction was carried out for checking the specificity of the samples using the Rotor-Gene Q cycler (Qiagen) and the ABsolute qPCR SYBR Green Mix from Thermo Fisher Scientific. A gene-specific primer pair (HSV-1 *us9* gene, custom made) was used for the test amplification. The PCR products were used for ONT library preparation and sequencing. The end-repair and adapter ligation steps were carried out as was described in the ‘Regular’ protocol, and in our earlier publication (Boldogkői et al., 2018). The ONT R9.4 SpotONFlow Cells were used for sequencing.

#### Application of Terminator Exonuclease

Some of the non-Cap-selected samples were treated by Terminator exonuclease (Epicentre/Lucigen) in order to reduce the proportion of sequencing reads with incomplete 5’-UTR regions. The protocol has been carried out as recommended by the manufacturer. Briefly, 2 µl of buffer A, 1 µg of total RNA, 0.5 µl of RNaseOUT (Invitrogen), and 1 U of Terminator exonuclease were mixed and incubated at 30°C for 60 min. The same reaction was carried out using buffer B instead of buffer A, after which the two mixtures were pooled.

### Oxford Nanopore Minion Platform—Direct RNA Sequencing

The ONT’s Direct RNA sequencing (DRS) protocol (version: DRS_9026_v1_revM_15Dec2016) and the ONT Direct RNA Sequencing Kit (SQK-RNA001) were used to examine the transcript isoforms without enzymatic reactions—to avoid the potential biases—as well as to identify possible base modifications alongside the nucleotide sequences. Polyadenylated RNA was extracted from the total RNA samples and it was subjected to DRS library preparation according to the ONT’s protocol (Boldogkői et al., 2018). The quantity of the sample was measured by Qubit 2.0 Fluorometer using the Qubit dsDNA HS Assay Kit (both from Life Technologies). The library was run on an ONT R9.4 SpotON Flow Cell. Basecalling was carried out using Albacore (v 2.3.1).

### Mapping and Data Analysis

The minimap2 aligner (Li, 2018) was used with options *-ax splice -Y -C5 –cs* for mapping the raw reads to the reference genome (X14112.1), followed by the application of the LoRTIA toolkit (https://github.com/zsolt-balazs/LoRTIA) for the determination of introns, the 5’ and 3’ ends of transcripts, as well as for detecting the full-length reads. Putative introns were defined as deletions with the consensus flanking sequences (GT/AG, GC/AG, AT/AC). The complete intron lists are available as additional material. We used even stricter criteria: only those splice sites were accepted, which were validated by dRNA-Seq [used in our present work and in Depledge and coworkers’ study (Depledge et al., 2019)]. These transcripts all have the canonical splice site: GT/AG and they are abundant (> 100 read in Sequel data).

The 5’ adapter and the poly(A) tail sequences were identified at the ends of reads by the LoRTIA toolkit based on Smith-Waterman alignment scores (**Table 2**). If the adapter or poly(A) sequence ended at least three nucleotides (nts) downstream from the start of the alignment, the adapter was discarded, as it could have been placed there by template-switching. Transcript features such as introns, transcriptional start sites (TSS) and transcriptional end sites (TES) were annotated if they were detected in at least two reads and in 0.1% of the local coverage. In order to reduce the effects of RNA degradation, only those TSSs were annotated, which were significant peaks compared to their ±50-nt-long windows according to Poisson distribution. Reads being connected a unique set of transcript features were annotated as transcript isoforms. Low-abundance reads detected in a single experiment were accepted as transcripts if the same TSS and TES were also used by other transcripts. In most cases, those reads were accepted as isoforms, which were detected in at least two independent experiments. The 5′-ends of the long low-abundance reads were checked individually using the Integrative Genome Viewer (IGV; https://software.broadinstitute.org/software/igv/download). The workflow of the data analysis can be found in **Supplementary Figure 1**.

**Table 2:** 5’ adapter sequences and settings for adapter detection with the LoRTIA pipeline. The scoring of the Smith-Waterman alignment was set to +2 for matches and -3 for mismatches, gap openings and gap extensions.

Method	Adapter sequence	Score limit	Distance from the start of the alignment
PacBio	AGAGTACATGGG	16	+5/–15
MinION	TGCCATTAGGCCGGG	15	+5/–15
Teloprime	TGGATTGATATGTAATACGACTCACTATAG	20	+5/–30

## Results

### Analysis of the HSV-1 Transcriptome With Full-Length Sequencing

In this work, we report the application of two distinct LRS techniques (the PacBio Sequel and the ONT MinION platforms), and multiple library approaches for the investigation of the HSV-1 lytic transcriptome. We also reutilized our previous PacBio RS II data for the validation of novel transcripts. The PacBio sequencing is based on an amplified Iso-Seq template preparation protocol that utilizes a switching mechanism at the 5’ end of the RNA template, and is thereby able to produce complete full-length cDNAs (Zhu et al., 2001). We applied both cDNA and dRNA sequencing for the ONT technique. Additionally, we used Cap-selection for a fraction of samples. A single sample was treated by Terminator exonuclease, which selectively degrades uncapped and non-polyadenylated transcripts. ONT sequencing was also used for the kinetic analysis of HSV-1 gene expressions. Sequencing reads were mapped to the HSV-1 (X14112) genome using the Minimap2 alignment tool (Li, 2018) with default parameters.

Altogether, we obtained 80,061 full-length ROIs mapping to the HSV-1 genome using Sequel sequencing, whereas PacBio RSII platform generated 38,972 ROIs (**Supplementary Table 1**). ONT sequencing produced altogether 1,505,848 sequencing reads mapping to the viral genome. The reason behind the relatively low proportion of the full-length read count within the MinION samples is that this method—compared to PacBio—generates a higher number of 5’ truncated reads. We and others have reported in previous publications that the dRNA-Seq method is not optimal for capturing entire transcripts (Moldován et al., 2017b; Moldován et al., 2018b; Workman et al., 2018): we found that short 5’ sequences of transcripts and in many cases the polyA-tails were missing from most of the reads. However, a recently published technique utilizing adapter ligation to the 5’ end of full-length mRNAs is able to solve this problem (Jiang et al., 2019). Another drawback of native RNA sequencing is its low throughput compared to cDNA sequencing. The advantage of dRNA-Seq is that it is free of false products which are typically produced by RT, PCR, and cDNA sequencing.

**Table 3** shows the average read lengths of mapped full-length ROIs and MinION reads in the different samples. A detailed description of reads obtained from all libraries is found in **Supplementary Table 1**.

**Table 3:** Average mapped read-lengths and transcript lengths.

Technique	Average length of the reads (bp)	Average length of the abundant full-length transcripts (bp)
PacBio RSII *oligo(dT)*	1,369	1,409
PacBio RSII *random*	924	NA
PacBio Sequel	1,923	1,789
ONT MinION 1D *oligo(dT)*	967	1,222
ONT MinION 1D *random*	766	NA
ONT MinION Cap-seq *oligo(dT)*	683	797
ONT MinION dRNA-Seq	823	NA
ONT MinION *Terminator*	873	1,225
ONT MinION *Cap-seq random*	388	NA
ONT MinION time points	826	1,232

The data obtained from the individual p.i. time-points are discussed in **Supplementary Table 1**.

Cap-selection performed suboptimally in our experiment, because it produced relatively short average sequencing reads. Random RT-priming allowed the analysis of non-polyadenylated transcripts and helped the validation of TSSs and splice sites. Additionally, this technique proved to be superior for identifying the 5’-ends of very long transcripts, including polycistronic and complex RNA molecules. Terminator exonuclease was used for the enrichment of intact TSSs of the transcripts.

The following technical artifacts can be generated by RT and PCR: template switching, and nonspecific binding of oligod(T) or PCR primers. In addition to poly(A) tails, oligo(dT) primers occasionally hybridize to A-rich regions of transcripts and thereby produce false reads. These products were discarded from further analysis, albeit in some cases we were unsure about the non-specificity of the removed reads. We ran altogether 11 parallel sequencing reactions using 8 different techniques for providing independent reads. Additionally, in some cases, the same TSS, TES or splice junctions were found in other transcripts detected within the same sequencing reaction which further enhanced the number of independent sequencing reads. In our earlier publication (Tombácz et al., 2017b), we could not detect all spurious products, therefore, in the present work, we have made a minor correction to our formerly published results.

We used a novel bioinformatics tool (LoRTIA) — developed in our laboratory — for the identification of TSS and TES positions, as well as splice donor and acceptor sites (**Supplementary Figure 1**). This software suite detected a total of 1,677 putative TSSs 162 putative TESs and 379 putative introns (**Supplementary Table 2**). A putative TSS or TES was accepted as real if LoRTIA detected it in at least three independent samples in the case of longer isoforms, and five independent samples in the shorter variants, including 5’-truncated ORF-containing RNAs. The reason for a more stringent selection criterion for the short isoforms is that these can be the result of fragmentation, which is not the case for longer isoforms. These analyses yielded altogether 537 TSSs and 77 TESs. Only those sequencing reads were accepted as transcripts, which contained a TSS and a TES annotated in the above way. This method yielded 667 transcripts (**Supplementary Table 3**). For very long transcripts (≥ 3,000 bp), we applied a different rule: a read was accepted as a transcript if it was longer than all annotated overlapping transcripts even if it was represented in a few copies and had no annotated TSS. A large number of very long transcripts were identified this way in most cases in the Sequel dataset. Thus, altogether 2,250 transcripts were identified in this study (**Supplementary Table 3**). We assume that much more low-abundance and very long transcripts are expressed by the HSV-1 genome than we detected with our very strict criteria. Our dataset is available for further investigations, which can confirm or reject these latter categories of putative transcripts.

For intron identification, we used the following criteria: the candidate intron had to carry one of the canonical splice junction sequences: GT/AG, GC/AG, AT/AC; and it had to be detected by dRNA-Seq and both cDNA-Seqs (PacBio and ONT platforms). Besides introns based on hard evidence, we enlist additional putative introns of which the criterion was their detection by both dRNA-Seq and at least one of the cDNA (PacBio or ONT) sequencings. The third category of introns includes very abundant splice variants and introns on very long transcripts that were exclusively identified using Sequel sequencing in most cases. This study identified a large number of rare variants with deletions, which represented less than 5% of the total isoforms of a certain transcript. These putative splice variants were not accepted as transcripts. Altogether, 182 introns were identified in terms of the above criteria, among which 155 carry canonical GT/AG, 22 GC/AG, and 2 AT/AC splice junction sequences (**Supplementary Table 2**). Our analysis detected 80 transcripts containing one or more of these introns (**Supplementary Table 3**).

### *In Silico* Analysis of Promoters and Poly(A) Signals

**Figure 1:**
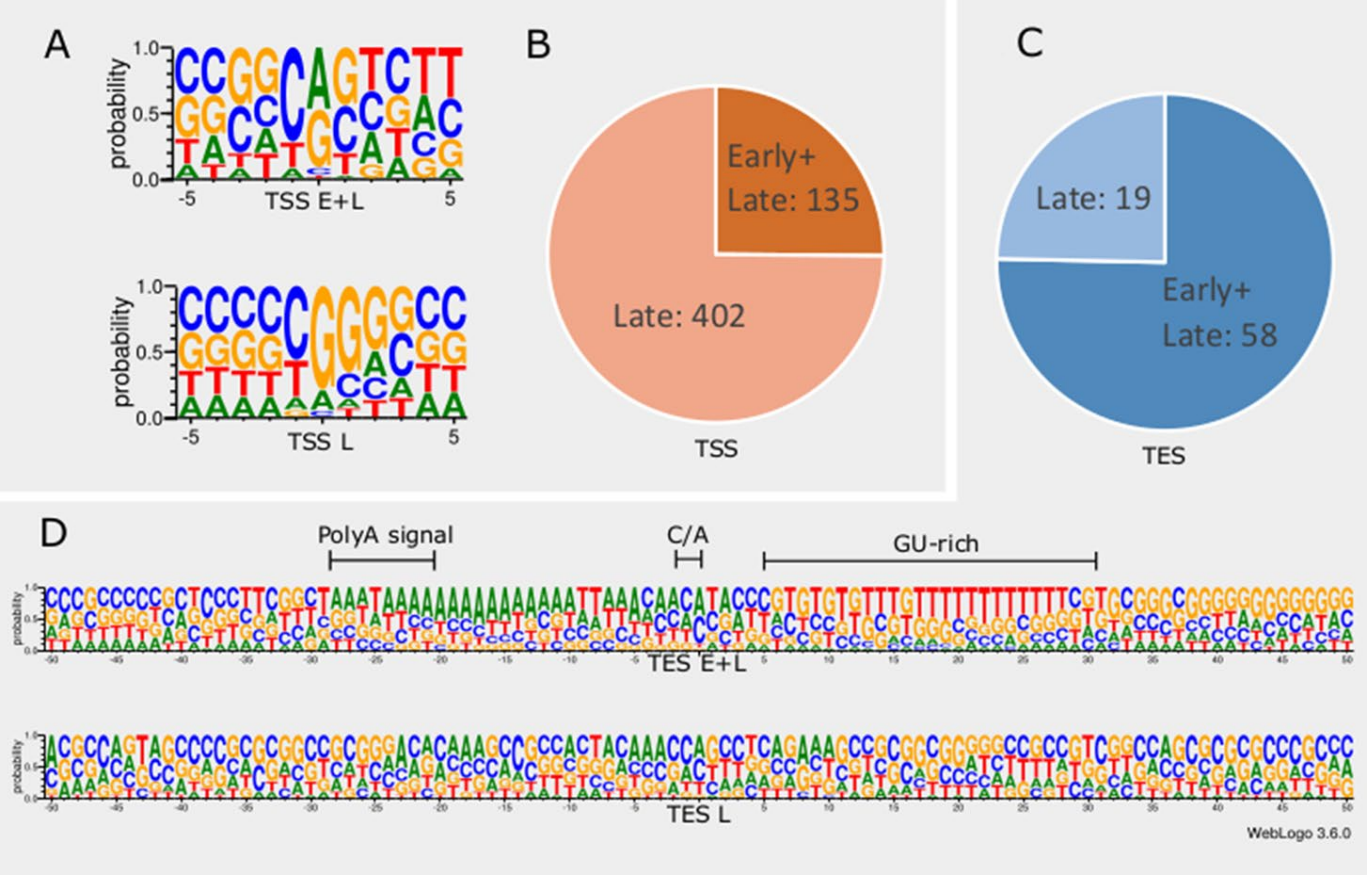
*In silico* analysis of INR and PAS sequences. **(A)** The initiator region (INR) of early samples is similar to the canonical eukaryotic INR sequence, while late INRs show homology with the VP5 promoter. **(B)** The proportion of TSSs present in both early and late or exclusively late time points of infection. **(C)** The proportion of TESs present in both early and late or exclusively late time points of infection. **(D)** The probability of expression of nucleotides in the ±50nt region of TESs throughout the entire infection period compared to those nucleotides that expressed only in late time points. TESs expressed during the entire period of infection (E+L) contain a canonical poly(A) signal, the C/A cleavage site and GU-rich downstream region. Late TESs lack a PAS and the canonical downstream elements, but they contain a GC-rich sequences 15-20nt downstream of the cleavage site.

In order to detect promoter sequences, we analyzed the -150 to +1 upstream region of the TSSs *in silico* (**Figure 1**). We found that 45% (371) of the TSSs are preceded by a canonical GC box sequence at a mean distance of 66.301nt (σ = 31.205), 4% (35) by a CAAT box at a mean distance of 113.428nt (σ = 15.471), and 11% (91) by a TATA box at a mean distance of 30.373nt (σ = 2.058) (Mackem and Roizman, 1982; Guzowski and Wagner, 1993). Some of the GC boxes may be nonfunctional, since they may be the result of the high GC-content of the viral genome. Earlier studies found a canonical initiator region (INR) ± 5 nt around the TSS of eukaryotic organisms (Lim et al., 2004; Xi et al., 2007). These have been shown to be used during the early viral gene expression, whereas late transcription is initiated from a G-rich sequence (Huang et al., 1996; Lieu and Wagner, 2000). We detected 16 TSSs containing a CAG INR (TSS position underlined) and 89 TSSs having YANW (Y: cytosine/thymine, N: adenine/cytosine/thymine/guanine, W: thymine/adenine, TSS position underlined).

We found that TSSs expressed in every time point are abundant and their INRs exhibit high similarity to canonical eukaryotic INRs, whereas TSSs from late samples are similar to the VP5 promoter (**Figure 1A**). Furthermore, these late TSSs are expressed in low abundance (2.8% of all reads starting in these positions) but their ratio is seven-fold higher than those of early TSSs (**Figure 1B**). We carried out *in silico* analysis of the -50nt region located upstream the TESs and detected 59 possible polyadenylation signals (PASs) at a mean distance of 21.779nt (σ = 5.558). The number of TESs expressed in both early and late phases is slightly higher than the number of TESs expressed only in the late phase of the viral life cycle (**Figure 1C**). TESs expressed throughout the entire viral replication are characterized by canonical PASs, cleavage signals and GU-rich regions. This is in contrast with TESs expressed only in the late phase, which tend to have no canonical signals for polyadenylation and cleavage (**Figure 1D**). Additionally, these late TESs are low abundance, representing only 0.1% of the reads’ 3’ ends.

### Novel Putative mRNAs

5’-Truncated transcriptional reads were accepted as transcripts if they were present in at least five independent samples. The first base had to be located within a ±5 window range. Additionally, reads having less than a 5% proportion at the overlapping region were discarded. Present investigations revealed 182 novel 5’-truncated mRNAs (tmRNAs) of HSV-1 (**Supplementary Table 4**), which were all produced from genes embedded in larger host genes of the virus. These 5’-truncated mRNAs are assumed to be generated by alternative transcription initiation from promoters located within larger genes. We could identify promoter modules for only 39 transcripts (we could not identify promoter consensus sequences for several canonical ORFs, too). These transcripts all contain in-frame ORFs. The first in-frame AUG triplet is assumed to encode the translation start codon. Further analyses have to be carried out to verify the coding potential of the ORF-containing tmRNAs. We detected a transcript — termed ‘RL-intron’ (RL2I) — with a TSS identical to that of the TSS of *rl2* gene but with a TES located within the intronic region of this gene. Our BLAST searches resulted in hypothetical proteins predicted to this ORF, but according to our knowledge, no such transcript has been detected until now.

### Novel Putative Non-Coding (or Coding) Transcripts

In this part of our study, we detected 18 putative non-coding RNAs, including antisense RNAs (asRNAs, termed as ASTs) and other putative long non-coding RNAs (lncRNAs) (**Table 4**). Furthermore, we validated and determined the base pair-precision termini of the transcripts published earlier by us and by others. **Supplementary Table 5** shows the potential peptides encoded by the ORFs on these transcripts. Further studies have to confirm whether these ORFs are translated. If so, they are novel protein-coding genes.

**Table 4:** Polyadenylated ncRNAs of HSV-1. **(A)** Previously detected and validated ncRNAs; **(B)** Novel ncRNAs. All transcripts are polyadenylated.

Name	Genomic locations	
**A**		
LAT 0.7 kb - S	7,643	8,393
LAT 0.7 kb	7,643	8,423
AST-1	57,711	59,429
AST-2-L4*	78,315	80,725
AST-2-L3*	78,531	80,725
AST-2 sp	79,792	80,725
AST-2	79,792	80,725
AST-3*	103,152	103,512
AST-4*#	110,816	112,131
LAT 0.7 kb	117,948	118,728
LAT 0.7 kb - S	117,978	118,728
**B**		
LAT 0.7 kb - ul1-2-3-3.5*	7,643	11,285
LAT 0.7 kb - S2	7,643	8,338
LAT 1.1 kb	7,643	8,733
AST-2-sp2	79,792	80,725
LAT 1.1 kb	118,033	118,728
LAT 0.7 kb - S2	117,638	118,728
LAT 0.7 kb - L*	115,083	118,728
AST-5	141,008	141,629

*unidentified 5’ end # unidentified 3’ end.

**Figure 2:**
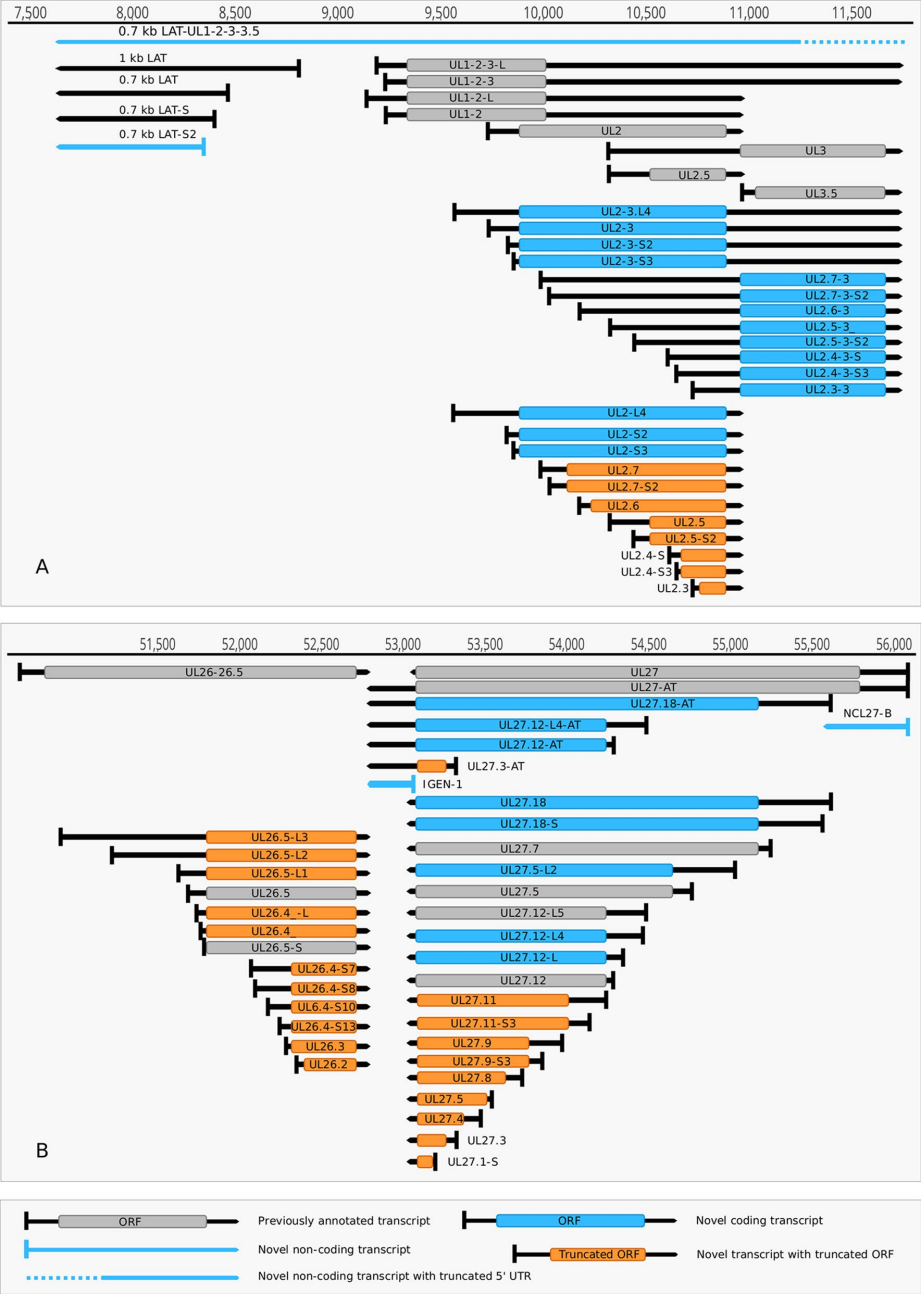
Non-coding HSV-1 RNAs. **(A)** Schematic representation of the LAT region and surroundings. Besides the previously published coding and non-coding transcripts, this figure illustrates the newly discovered shorter TSS version of the 0.7 kb LAT, as well as the oppositely oriented transcript isoforms, which are co-terminal with the 3’ ends of the UL2 or UL3 transcripts. **(B)** A novel non-coding transcript designated IGEN-1 is co-terminal with UL27-AT which is a longer TES isoform of UL27. Several other 5’ UTR length variants were discovered and annotated in the UL26-UL27 region.

**(1) Antisense RNAs** These transcripts can be controlled by their own promoters or by the promoter of another (mRNA) gene. It has earlier been reported that the 0.7-kb LAT transcript is not expressed in strain KOS of HSV-1 (Zhu et al., 1999). Here we demonstrate that this is not the case, since we were able to detect this transcript. The existence of the shorter LAT-0.7kb-S (Tombácz et al., 2017b) was also confirmed. Additionally, we detected asRNAs being co-terminal with the LAT-0.7 transcripts, but having much longer TSSs. The LAT region and its surrounding genomic sequences are illustrated in **Figure 2A**. Using random oligonucleotide-based LRS techniques, we obtained a large number of antisense-oriented reads, most of them without identified 5’-ends. We also detected antisense transcripts without defined TSSs and TESs within 27 HSV-1 genes (*rl1, rl2, ul1, ul2, ul4, ul5, ul10, ul14, ul15, ul19, ul23, ul29, ul31, ul32, ul36, ul37, ul39, ul42, ul43, ul44, ul49, ul50, ul53, ul54, us4, us5, us8*). The expression level of these asRNAs is low, in most cases only a few reads were detected *per* gene locus. However, a high level of antisense RNA expression was identified within the locus of *ul10* gene. A special class of asRNAs is produced by divergent genes, and read-through RNAs (rtRNAs) generated by transcriptional read-through between convergent gene pairs. These transcripts are mRNAs with long stretches of antisense segments. For example, we detected an antisense transcript originated within the 3’ region of *ul4* gene and co-terminated with UL6-7 bicistronic transcript. This RNA molecule contains three splice sites, and can be considered as a very long TSS isoform of the UL6-7 transcript.

**(2) Intergenic ncRNAs** A ncRNA (termed “intergenic ncRNA”; IGEN-1) located between the *ul26* and *ul27* genes was also identified. This transcript is co-terminal with the UL27-AT RNA, which is a longer TES isoform of UL27 transcript (**Figure 2B**). Another non-coding transcript (IGEN-2) with unidentified transcript ends was detected to be expressed in the outer termini of the HSV-1 unique long region. The potential function of IGEN transcripts remains unclear. A bidirectional, low-level expression from the intergenic region between the *rl2* (icp0) and LAT genes was also observed. These RNA molecules are co-terminal with the LAT-0.7kb transcript and may be parts of the potential RL2-LAT-UL1-2-3 transcript (Tombácz et al., 2017b). Additionally, we detected RNA expression in practically every intergenic region.

**(3) Intra-intronic ncRNAs** A ncRNA was identified within the intron of the *rl2* gene, which was designated as NCIRL2. This transcript is expressed in a low abundance.

### Replication-Associated Transcripts

**Figure 3:**
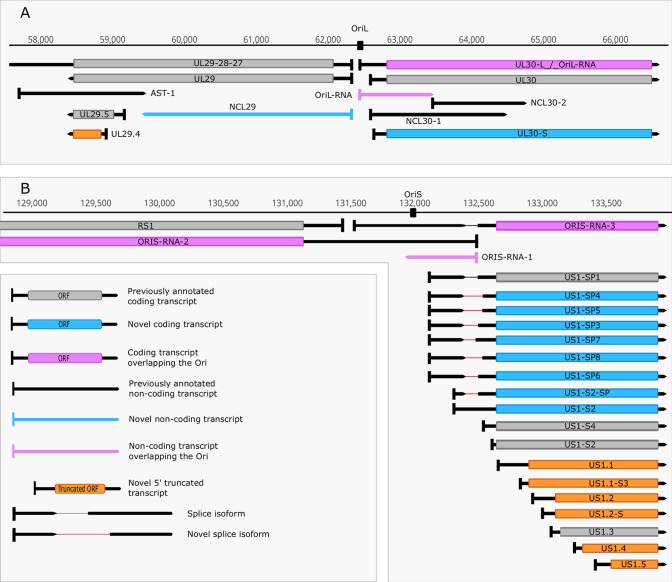
Replication associated transcripts of HSV. **(A)** A novel shorter 5’-UTR isoform of the UL30, and a non-coding transcript sharing the TSS with UL29 but terminating within its ORF was discovered in the vicinity of Ori-L. **(B)** Two isoforms with shorter 5’-UTRs, seven splice isoforms and six novel putative protein-coding transcripts were annotated downstream of Ori-S.

We identified five replication-associated RNAs (raRNAs) designated OriL-RNA1-2, and OriS-RNA1-3, which overlap the replications origins OriL and OriS, respectively. OriL-RNA1 is a long TSS isoform produced from the *ul30* gene, whereas OriS-RNA2 is a TSS variant of *rs1* (*icp4*) (**Figure 3**). OriL-RNA2 is a transcript without an annotated TES. We suppose that this transcript is the long TSS variant of the *ul29* gene. We were only able to detect certain segments but not the entire OriS-RNA1 described by Voss and Roizman (1988). We also detected a longer TSS isoform of the *us1* gene (US1-L2 = OriS-RNA3) which overlaps the OriS located within the terminal repeat of US region (TRS) (**Figure 3**). Additionally, OriS is also overlapped by a longer 5’ variant of the *us12* gene (US12-11-10-L2 = OriS-RNA-4).

### TSS and TES Isoforms

**Figure 4:**
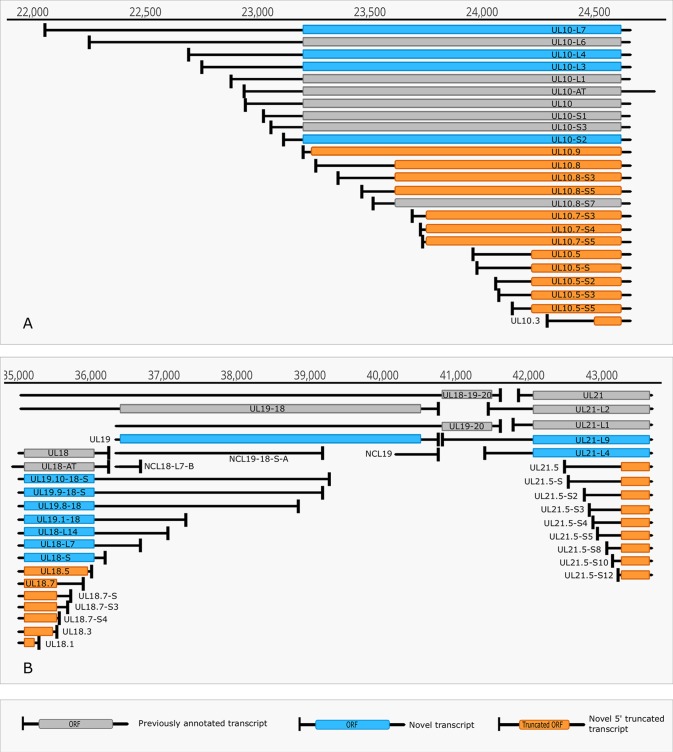
Complexity of TSSs. **(A)** The TSS pattern of UL10 transcript exhibits an especially high complexity. Several TSSs are located downstream from the translation initiation site, resulting in truncated ORFs. RNAs harboring these truncated ORFs may code for N-terminally truncated transcripts or may be non-coding RNA. **(B)** Divergent overlaps between the ul20 and ul21 genes. These overlaps are caused by the high variability in the TSS of UL21.

The multiplatform system allowed the discovery of novel RNA isoforms and reannotation of the transcript termini published earlier by others and us (Tombácz et al., 2017b; Depledge et al., 2019). The LoRTIA software suit — used for the detection of TSS and TES positions — identified 218 TSS and 56 TES positions (**Supplementary Table 2**). Altogether 53 genes produce at least one TSS isoform, besides the most frequent variants (**Supplementary Table 3**). Fifteen genes were found to produce three different transcript length isoforms (including the most frequent versions). The recent LRS analysis discovered 51 protein-coding and 2 (0.7 kb LAT, and RS1) non-coding transcripts with alternative TSSs. However, a few transcripts with unannotated 5’-ends were also detected (**Supplementary Table 3**). The alternative TSSs may lead to transcriptional overlap or they may enlarge the extent of existing overlaps especially between divergently transcribed genes. Some transcripts (e.g. UL19 and UL10) exhibit an especially high complexity of TSS isoforms (**Figure 4A**). The *ul21* gene produces nine different 5’ length variants, the longer ones overlap the divergently oriented *ul22* gene) (**Figure 4B**). Additionally, long TSS isoforms are responsible for the overlaps of each replication origin of HSV-1, which is not the case in PRV, its close relative (Tombácz et al., 2015; Boldogkői et al., 2019a). Many of the longer TSS variants contain upstream ORFs (uORFs), which may carry distinct coding potentials as described by Balázs and colleagues in the human cytomegalovirus (Balázs et al., 2017a). Two novel 3’-UTR variants were also identified in this study.

### Novel Splice Sites and Splice Isoforms

**Figure 5:**
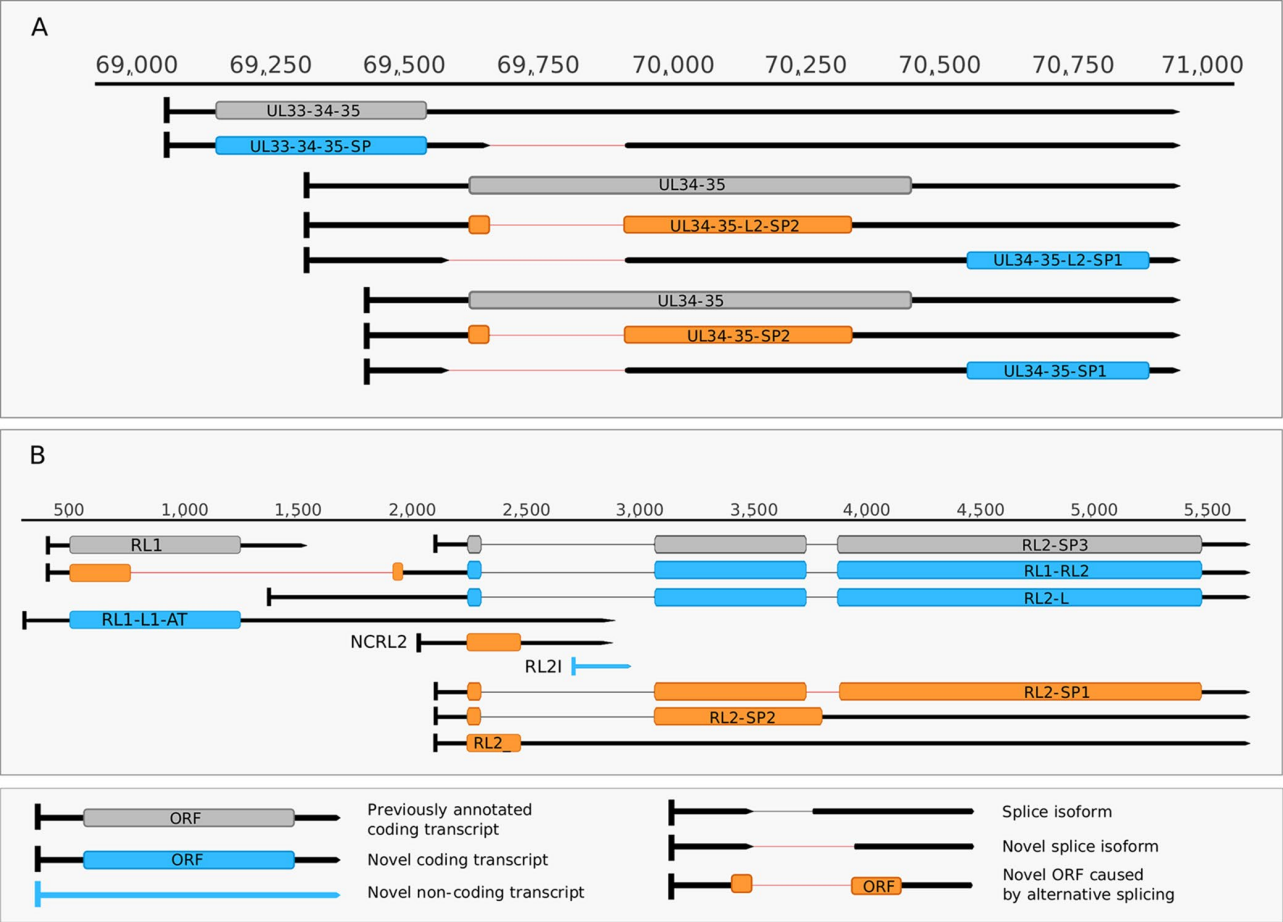
Complexity of spliced transcripts. **(A)** The splice sites of UL34-35 transcript were confirmed by dRNA sequencing. The splicing event leads to frameshifting of the ul34 ORF in UL34-35-SP2 and UL34-35-L-SP2 and in the deletion of the translational initiation site of the ul34 ORF in UL34-35- SP1 UL34-35-L2-SP1. **(B)** The splicing complexity of RL2 and novel non-coding and potentially coding transcripts overlapping RL1 and the 5’ UTR of RL2. Alternative splicing in RL2-SP1 produces a frameshift in the second half of the ORF, while alternative splicing in RL2-SP2 and in RL2 results in premature stop codons. Two novel 5’-UTR length isoforms of the RL2 transcripts are shown, one of which may contain a truncated form of the RL1 ORF. This truncation is caused by a splicing event. A novel isoform of the RL1 with longer 5’ and 3’ UTRs (designated as RS1-L1-AT) was discovered. Another novel putative protein coding transcript is the RS2-ALT, which is co-terminal with RS1-L1-AT.

In this study, we also used dRNA sequencing, which provides a fundamentally different method from cDNA sequencing and hence can be utilized to filter out spurious splice sites. The splice donor and acceptor sites were also detected by using the LoRTIA tool. Altogether, using different sequencing techniques and bioinformatics analyses, we were able to verify the existence of 5 previously described and 30 novel splice sites. **Table 5** contains the list of introns, which were confirmed by dRNA-Seq (**Figure 5**). By far the most complex splicing pattern was detected in RNAs produced from the *ul41-45* genomic region.

**Table 5:** The most frequent splice sites of the HSV-1 transcriptome.

Intron start	Intron end	Read count	DNA strand	Splice donor/acceptor	
2,318	3,082	20	+	GT/AG	
3,750	3,888	6	+	GT/AG	*
3,750	3,885	8	+	GT/AG	
13,449	13,931	37	–	GT/AG	*
30,049	33,634	198	+	GT/AG	
69,593	69,923	12	+	GT/AG	*
69,670	69,923	20	+	GT/AG	*
71,622	71,712	2	–	GC/AG	*
71,622	71,718	6	–	GC/AG	*
71,622	71,724	2	–	GC/AG	*
71,622	71,736	2	–	GC/AG	*
71,622	71,748	4	–	GC/AG	*
91,553	92,535	120	+	GT/AG	
97,724	97,949	228	+	GT/AG	
113,428	113,786	40	+	GT/AG	*
122,483	122,621	7	–	GT/AG	*
122,486	122,621	8	–	GT/AG	*
123,289	124,053	20	–	GT/AG	*
132,373	132,540	74	+	GT/AG	*
132,373	132,506	269	+	GT/AG	*
132,373	132,487	34	+	GT/AG	*
132,373	132,543	2	+	GT/AG	*
132,381	132,518	2,995	–	GT/AG	*
132,386	132,540	11	+	GT/AG	*
132,386	132,506	34	+	GT/AG	*
132,386	132,509	31	+	GT/AG	*
145,646	145,820	66	–	GT/AG	*
145,646	145,860	34	–	GT/AG	*
145,649	145,820	1,077	–	GT/AG	*
145,649	145,860	824	–	GT/AG	*
145,649	145,847	3	–	GT/AG	*
145,671	145,852	23	+	GT/AG	*
145,671	145,873	13	+	GT/AG	*
145,680	145,847	7	–	GT/AG	*
145,683	145,860	53	–	GT/AG	*
145,683	145,847	17	–	GT/AG	*

The newly discovered splice sites are labeled with asterisks.

### Novel Multigenic Transcripts

Our earlier survey has revealed several novel multigenic RNAs, including polycistronic and complex transcripts (Tombácz et al., 2017b). In this work, we identified 201 multigenic transcripts containing two or more genes (**Supplementary Table 3**). The cxRNAs are long RNA molecules with at least 2 genes standing in opposite orientation relative to one another. Our intriguing findings are the RL1-RL2 (ICP34,5-ICP0) bicistronic transcript, as well as the 0.7. kb LAT-UL1-2-3-3.5 cxRNA (**Figures 2A, B**). Most of the novel multigenic transcripts are expressed at low levels, which can explain why they had previously gone undetected. In this work, we also identified four novel complex transcripts (0.7 kb LAT-UL1-2-c, UL18-15.5-c, UL20-21-c, US4-3-2-c) with unannotated TSSs (**Figure 2A**). We were able to detect these transcripts by cDNA sequencing and by the reanalysis of a MinION dRNA sequencing dataset (Depledge et al., 2019). Our novel experiments validated previously published cxRNAs. This study demonstrates that full-length overlaps between two divergently-oriented HSV-1 genes are an important source for the cxRNA molecules. The likely reason for the lack of cxRNA TSSs in many cases is that they are very long and low-abundance transcripts. It cannot be excluded with absolute certainty that some of the low-abundance multigenic transcripts are artefacts produced by the template–switch mechanism; other approaches are needed for the validation of their existence one-by-one.

### Novel Transcriptional Overlaps

**Figure 6:**
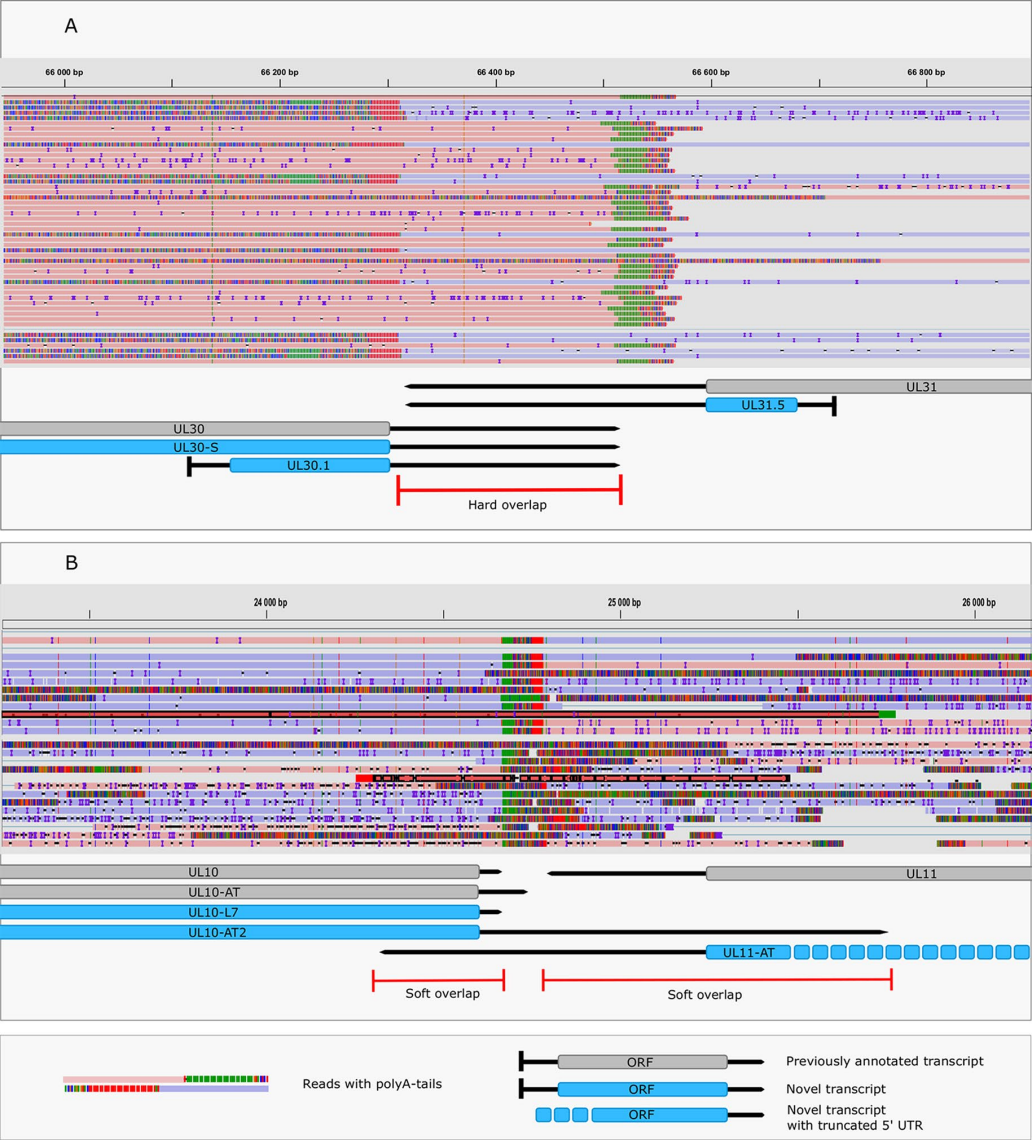
Transcriptional overlaps. **(A)** A hard convergent overlap between the 3’-UTR regions of UL30 and UL31 transcripts shown by sequencing reads and annotations. **(B)** Occasional overlapping events between UL10-AT2 and UL11 and between UL11-AT and UL10 termed “soft convergent overlap”. The reads representing UL10-AT2 and UL11-AT are shown in dark red. Reads were visualized using IGV.

This study revealed an immense complexity of transcriptional overlaps (**Figure 6** and **Table 6**). These overlaps are produced by either transcriptional read-through events between transcripts oriented in parallel [as described in Kara et al. (2019)], or in a convergent manner (thereby generating rtRNAs), or through the use of long TSS isoforms pertaining to one or of both partners of divergently-oriented genes. Transcriptional overlaps can also be produced by antisense transcripts controlled by their own promoters, as seen in LAT transcripts. Besides the ‘soft’ (alternative) overlaps, adjacent genes can also produce ‘hard’ overlaps when only overlapping transcripts are produced from the same gene pairs. An important novelty of this study is the discovery that practically each convergent gene produces rtRNAs crossing the boundaries of the adjacent genes. Two of the convergent gene pairs (*ul3*-*ul4* and *ul30*-*ul3*1) form ‘hard’ transcriptional overlaps, whereas the other gene pairs form ‘soft’ overlaps. The ‘softly’ overlapping convergent transcripts are likely to be non-polyadenylated, since we were only able to detect most of them by the random primer-based sequencing technique. The *ul3-ul4* and *ul30-31* gene pairs also express non-polyadenylated rtRNAs that extend beyond their poly(A) sites. Transcriptional read-troughs were detected between each convergent gene pair in most cases from both directions, except in the UL43-44-45/UL48-47-46 cluster (**Figure 6** and **Table 6**). Another important novelty of this study is the discovery of very long TSS variants of divergent transcripts, the 5’-UTRs of which entirely overlap the partner gene. We detected very long transcripts which overlap the following divergent gene clusters: *ul4-5/ul6-7, ul4-5/ul6-7, ul4-5/ul6-7, ul4-5/ul6-7, ul9-8/ul10, ul9-8/ul10, ul14-13-12-11/ul15, ul17/ul15e2, ul20-19-18/ul21, ul20-19-18/ul21, ulL23-22/ul24-25-26, ul29/*OriL*/ul30, ul29/*OriL*/ul30, ul32-31/ul33-34-35, ul37/ul38-39-40, ul41-ul42, ul49.5.49/ul50, ul51/ul52-53-54, us2/US3, us2/us3, us2/us3*. Altogether, our results show that practically every nucleotide of the double-stranded HSV-1 DNA is transcribed.

**Table 6:** Read-through RNAs. **(A)** Novel ncRNAs with unidentified 3’ ends; **(B)** Novel ncRNAs with unidentified 5’ and 3’ ends.

Name	Genomic locations	
**A**		
rtUL3-4	11,212	12,316
rtUL8-7	17,579	18,659
rtUL16-15L1	30,000	31,607
rtUL51-S-50	107,877	109,169
rtUL51-50	108,179	109,305
rtUL56-55-54-c	114,529	117,080
rtUS2-US1	133,243	135,306
rtUS1-US2	132,127	135,322
rtUS11-10-9	143,185	145,461
rtUS12-11-10-9	143,752	146,102
**B**		
IGEN-2 (earlier name: ULTN)	6,154	6,608
rtUL4-UL3	11,697	12,500
rtUL7-8	17,931	19,042
rtUL15-18	29,241	35,597
rtUL18-15	34,818	35,068
rtUL21-22	42,780	45,087
rtUL22-21/1	41,950	44,076
rtUL22-21/2	43,654	46,359
rULl26-27	52,662	54,774
rtUL36-35	71,000	71,520
rtUL41-40	89,898	91,274
rtUL40-41	90,900	91,712
AST-3-L	101,939	103,511
AST-3-UL49.5 rtRNA	102,801	103,952

These rtRNAs are probably non-polyadenylated because most of them were detected by random-primed sequencing alone. The genomic locations indicate the mapping of the transcription reads and not the transcript termini. “rt” stands for “read-through”, “c” for “complex”.

### Kinetics of HSV-1 Transcripts

**Figure 7:**
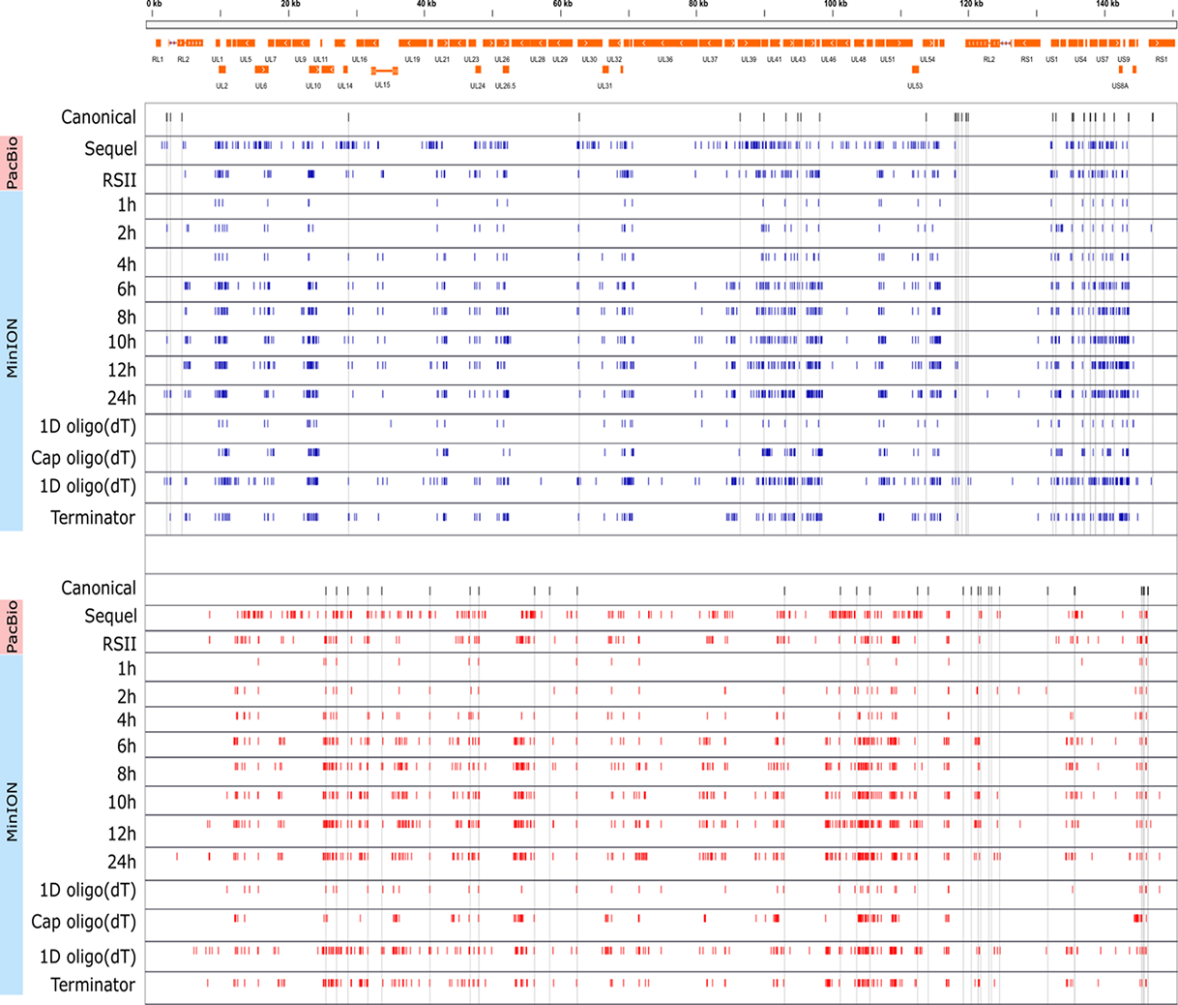
Genome-wide kinetics of the TSSs of HSV-1. The TSSs were determined using the LoRTIA software suite in each sample. Blue dashes represent TSSs on the forward strand, while red dashes represent TSSs on the reverse strand. Black dashes represent previously known TSSs, whereas grey lines starting from the TSS and spanning to the bottom of the figure show the locations of known TSSs in every sample. Orange rectangles represent the ORFs. A higher resolution illustration is presented in **Supplementary Figure 2**.

**Figure 8:**
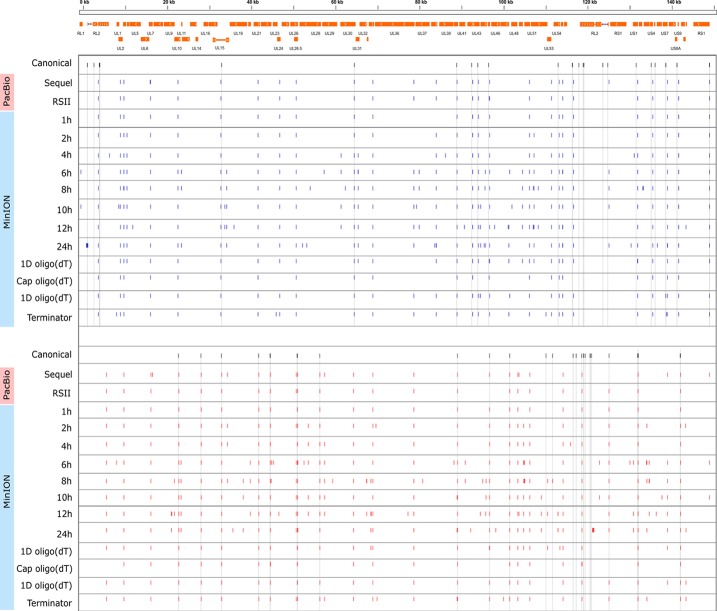
Genome-wide kinetics of the TESs of HSV-1. The TESs were determined using the LoRTIA software suite in each sample. Blue dashes represent TESs on the forward strand, while red dashes represent TESs on the reverse strand. Black dashes represent previously known TESs, whereas grey lines starting from these and spanning to the bottom of the figure show the locations of known TESs in every sample. Orange rectangles represent the ORFs.

**Figure 9:**
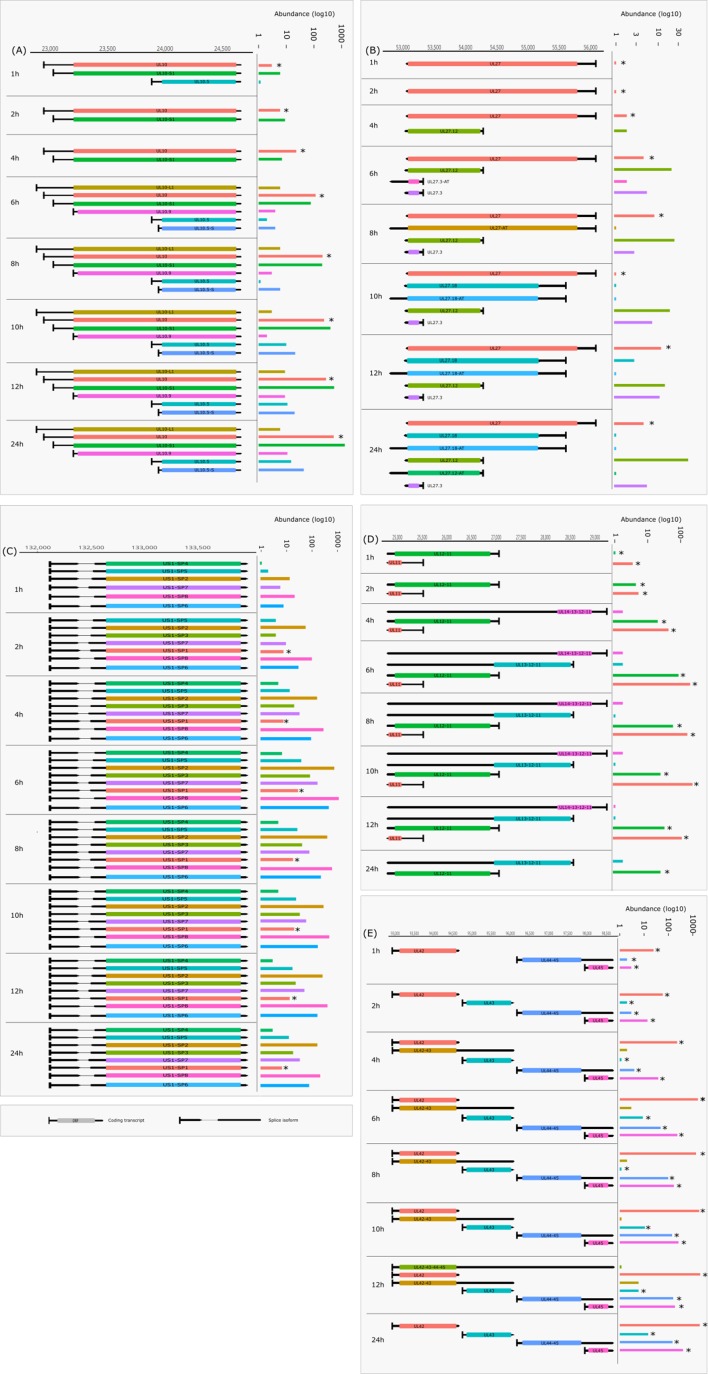
Dynamic HSV-1 transcriptome—examples. The structure of transcript isoforms and of their position on the HSV-1 genome is shown by the annotations, while their abundance in distinct time points of the infection is represented on a log10 scale by bar plots on the right side of the annotation. Transcripts annotated in other works are marked with an asterisk (*). Transcript structures and counts were determined using the LoRTIA software suite. **(A)** The change in abundance of the 5’-UTR and 5’ truncated isoforms of UL10. **(B)** Expression of UL27 RNA and its isoforms, including those with alternative termination. **(C)** Transcription kinetics of the US1 splice variants. **(D)** The change in abundance of polycistronic and monocistronic transcripts in the coterminal transcript at the UL11-UL14 region. **(E)** Transcription kinetics abundance of polycistronic and monocistronic transcripts in the UL42-UL45 region. Some of these transcripts are coterminal, while others have alternative terminations.

Cultured Vero cells were incubated with HSV-1 for 1, 2, 4, 6, 8, 10, 12, or 24 h. Altogether, we obtained 1,028,840 viral reads in the kinetic part of the study (**Supplementary Table 1**). The distribution of TSSs and TESs along the HSV-1 genome is illustrated in **Figure 7** (see in detail in **Supplementary Figure 2**) and **Figure 8**. The dynamics of various transcript categories is exemplified in **Figure 9**, including tmRNAs (**panel A**), TSS isoforms (**panel B**), TES isoforms (**panel C**), splice variants (**panel D**), and polycistronic RNAs (**panel E**). Many mono- and polycistronic RNAs and transcript isoforms are differentially expressed throughout the replication cycle of the virus. The cumulative abundance of transcript isoforms in distinct period of HSV infection is depicted in **Supplementary Figure 3**.

## Discussion

In the last couple of years, LRS approaches revealed that the viral transcriptome is substantially more complex than previously thought (Boldogkői et al., 2019b). In this study, 2 sequencing platforms (PacBio Sequel and ONT MinION) and 8 library preparation methods were applied for the investigation of the HSV-1 lytic transcriptome, including both poly(A)^+^ and poly(A)^-^ RNAs. This research yielded a number of novel transcripts and transcript isoforms. We identified novel tmRNAs embedded into larger host viral genes. All of these short novel transcripts contain in-frame ORFs, but it does not necessarily mean that this coding potency is realized in translation. Indeed, most of the putative tmRNAs are expressed in low abundance (these were not accepted as transcripts), which raises doubts as to whether they code for proteins. These transcripts might have a regulatory role in certain step(s) of gene expression, but we cannot exclude that they represent mere transcriptional noise.

This study also identified a large number of transcript length isoforms varying in their TSSs or TESs. In certain genes, we obtained very high number of TSS isoforms, therefore we did not name them individually. Many of these length variants are expressed in low abundance. It is unknown whether these transcripts have distinct roles, or their function is exactly the same as the high-abundance variants. It is possible that increasing coverage further would reveal that transcripts are initiated from a promoter at each nucleotide within a certain stretch of DNA with varying probabilities. In the human cytomegalovirus and HSV it has been shown that the longer TSS variants may contain uORFs which may have a role in the translational regulation of downstream ORFs, and shorter TSSs, on the other hand, often contain N-terminally truncated ORFs (Stern-Ginossar et al., 2012; Balázs et al., 2017a; Whisnant et al., 2019).

In this work, we also detected novel splice sites and splice isoforms. We applied very strict criteria for the identification of introns, therefore, many low-abundance introns have been eliminated. Indeed, after the submission of our manuscript, Tang et al. (2019) have reported the existence of several hundreds of splice sites in HSV-1. Further studies have to decide whether these putative introns are artifacts or they really exist.

Here, we also report the identification of several multigenic RNA molecules including polycistronic and complex transcripts. The existence of cxRNAs, expressed from convergent gene pairs, indicates that transcription does not stop at gene boundaries but occasionally continues across genes standing in opposite directions of one another. The cxRNAs are typically expressed in low amount: however, their abundance is difficult to determine precisely because the amount of long transcripts is significantly underestimated by LRS techniques.

We have also detected pervasive antisense transcript expression throughout the entire viral genome especially with the random primer-based sequencing method. Novel antisense RNAs are typically transcriptional read-through products specified by the promoter of neighboring convergent genes. These normally low-abundance, non-polyadenylated transcription reads contain varying 3’ends. The reason of this phenomenon is the use random nucleotide primers for the RT. The HSV-1 genome also expresses antisense RNAs controlled by their own promoters. For example, we identified a very long 5’-UTR isoform of LAT-0.7 transcript. The LAT RNAs have been shown to play a role in latency (Nicoll et al., 2016). LAT has also been shown to be a source of miRNAs (Lieberman, 2016). Further studies are needed to establish the potential function of LAT expression during the lytic cycle. We also detected novel divergent transcriptional overlaps: in two cases these transcripts appear to be initiated from the 3’-ends of the adjacent genes.

In another article, we proposed a potential function for the complex overlapping meshwork formed by transcriptional read-throughs, divergent overlaps, antisense RNAs, as well as polygenic transcripts. We suggest the existence of a novel regulatory layer based on genome-wide interactions between closely located genes through the collision of and competition between their transcriptional machineries (Boldogkői et al., 2019c).

Moreover, we could also identify 2 novel replication-associated transcripts—OriL RNA-1 and OriS RNA-3—overlapping OriL and OriS, respectively. Both raRNAs are long TSS isoforms produced from the neighboring genes, *us1* for OriS, and *ul30* for OriL. Similar transcripts have also been recently described in other alphaherpesviruses (Moldován et al., 2017b; Boldogkői et al., 2018; Prazsák et al., 2018). Intriguingly, since the replication origin is located at different genomic regions of herpesviruses, the sequences of raRNAs are non-homologous. The function of these transcripts may be the regulation of the initiation of replication fork as in bacterial plasmids (Tomizawa et al., 1981; Masukata and Tomizawa, 1986), or the regulation of replication orientation through a collision-based mechanism, as suggested earlier (Tombácz et al., 2015; Boldogkői et al., 2019a). In the latter case, raRNAs are mere byproducts of a regulatory mechanism, but it does not exclude the possibility that these transcripts have their own functions, which are at least partly different from those of shorter isoforms.

The analysis of the HSV-1 dynamic transcriptome has revealed a temporally differential expression of transcript isoforms, which suggests a function of these forms of diversity.

The prototypic organization of herpesvirus transcripts with respect to the location of genes is as follows (in the case of adjacent genes): abcd, bcd, cd, and d. However, there are some exceptions to this rule, e.g. the *ul41-43* and *ul51-49* regions. Both the regular and the irregular gene clusters exhibit time course differences in their location in mono- and various polycistronic RNAs. Genes are also transcribed in various combinations on RNA molecules but the expression of most genes follows the prototypic organization. All in all, this study identified several novel RNA molecules, and transcript isoforms. Further studies have to be carried out to ascertain the function of these transcripts. The question might be raised as to whether the low-abundance transcripts have any function at all, or whether they are the product of transcriptional noise. These transcripts may also be the by-products of a genome-wide regulatory mechanism discussed above, or they may also be functional.

## Accession Number

The PacBio RSII sequencing files and data files have been uploaded to the NCBI GEO repository and can be found with GenBank accession number GSE97785. The alignment files from MinION pooled samples, individual time points and Sequel sequencing have been deposited to the European Nucleotide Archive (ENA) under accession number PRJEB25433. Additional data from other sources utilized in this work for validation of rare transcripts and isoforms are available at the ENA with the study accession code PRJEB27861 (MinION dRNA-seq).

## Data Availability

The datasets generated for this study can be found in European Nucleotide Archive, PRJEB25433.

## Author Contributions

DT designed the experiments, prepared the PacBio and ONT sequencing libraries, performed the PacBio RSII, Sequel and ONT MinION sequencing, analyzed the data, and drafted the manuscript. NM analyzed the dynamic transcriptome data and drafted the manuscript. ZBa adapted the LoRTIA pipeline for the analysis. GG analyzed the PacBio and ONT dataset and maintained the cell cultures. ZC isolated RNAs, generated cDNAs, prepared ONT libraries, and performed ONT MinION sequencing. MB analyzed the PacBio data and made manuscript revisions. MS conceived and designed the experiments. ZBo conceived and designed the experiments, supervised the study, analyzed the data, and wrote the final manuscript. All authors have read and approved the final version of the manuscript.

## Funding

This study was supported by OTKA K 128247 to ZBo and OTKA FK 128252 to DT. DT was also supported by the Bolyai János Scholarship of the Hungarian Academy of Sciences and by the Eötvös Scholarship of the Hungarian State. The project was also supported by the NIH Centers of Excellence in Genomic Science (CEGS) Center for Personal Dynamic Regulomes [5P50HG00773502] to MS.

## Conflict of Interest Statement

The authors declare that the research was conducted in the absence of any commercial or financial relationships that could be construed as a potential conflict of interest.

The handling editor declared a past collaboration with several of the authors ZB, MS.

## Supplementary Material

Supplementary Figure 1 Workflow of the data analysis.

Supplementary Figure 2 High resolution TSS kinetics. TSSs and TESs were determined using the LoRTIA software suite in each sample. Blue dashes represent TSSs on the forward strand, while red dashes represent TSSs on the reverse strand. Orange rectangles represent the ORFs.

Supplementary Figure 3 The cumulative abundance of transcript isoforms. Transcript isoforms were annotated and counted in separate stages of the viral infection using the LoRTIA software suite. The names of isoforms annotated in previous works by other methods are in red, whereas the isoforms detected by long-read sequencing are in black.

Supplementary Table 1 Reads’ statistics.

Supplementary Table 2 TSSs, TESs and introns.

Supplementary Table 3
**(A)** Genome coordinates and abundance of transcripts identified by software. TSSs with bold letters were detected in at least 3 independent samples. **(B)** Spliced transcripts with genome coordinates and intron abundances. Abbreviations: HA: highly abundant, A, abundant; LA, low abundance.

Supplementary Table 4 Novel 5’-truncated transcripts with putative coding potential. This table summarizes novel and the previously published embedded mRNAs, as well as their genomic positions. Asterisks indicate transcripts that were also detected in our earlier study (Tombácz et al., 2017b).

Supplementary Table 5 NcRNA_codepot table. The table enlists the transcript start and end positions, the ORF composition, excluding introns for spliced ORFs, the orientation of the ORFs, the size of the ORF and the amino acid sequence of the ORF. Homology of these ORFs was analyzed by aligning them to Non-redundant protein database using the BLASTp suite. Hits with the highest E-score were included in the table.
