# Supplementary figures and images for: Multiple Long-Read Sequencing Survey of Herpes Simplex Virus Dynamic Transcriptome
Source: Front Genet. 2019 Sep 24;10:834. doi: 10.3389/fgene.2019.00834 (PMC6769088; doi:10.3389/fgene.2019.00834)

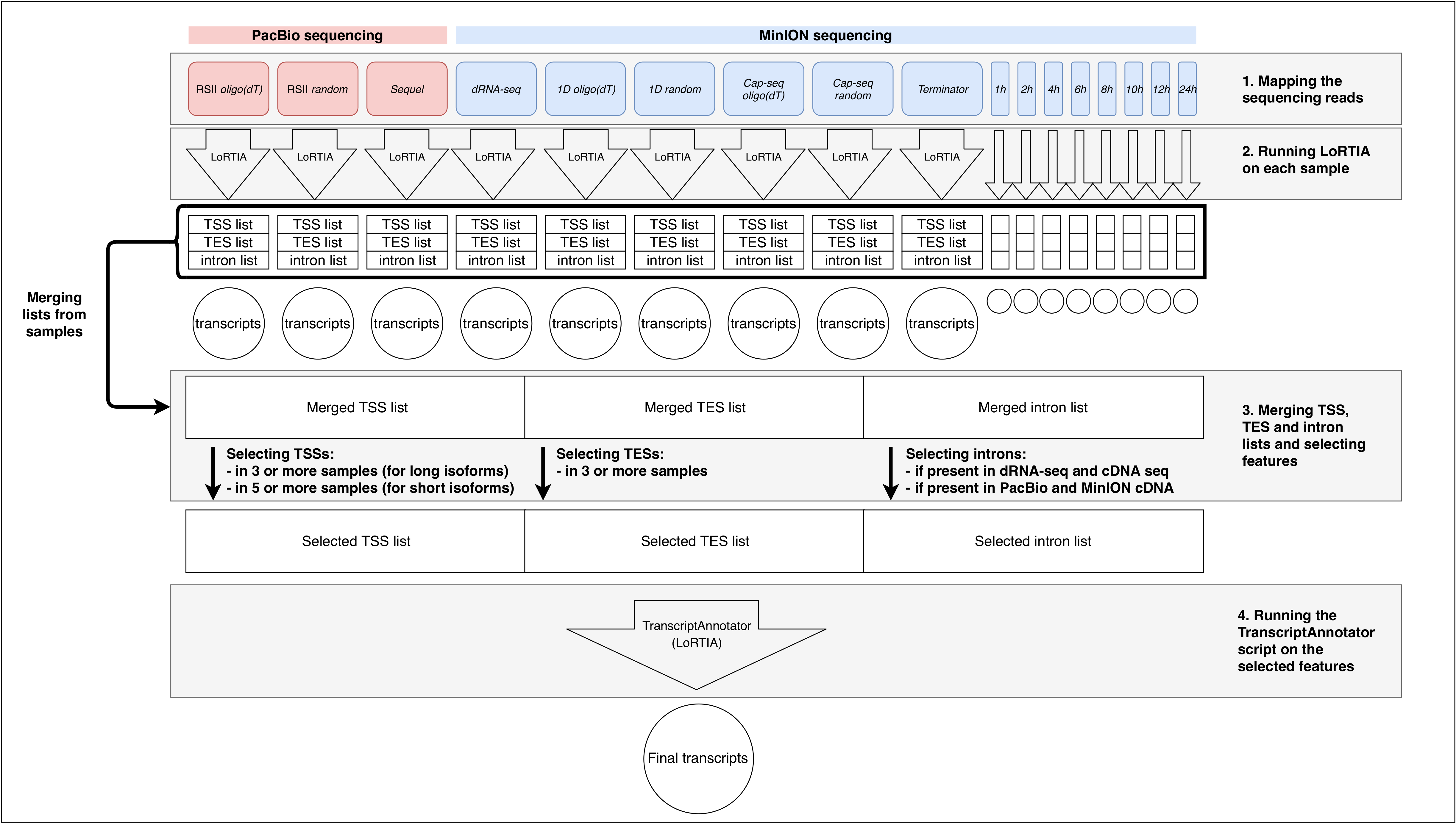

Supplement: Supplementary Figure 1 — Workflow of the data analysis. [file Image_1.tiff]

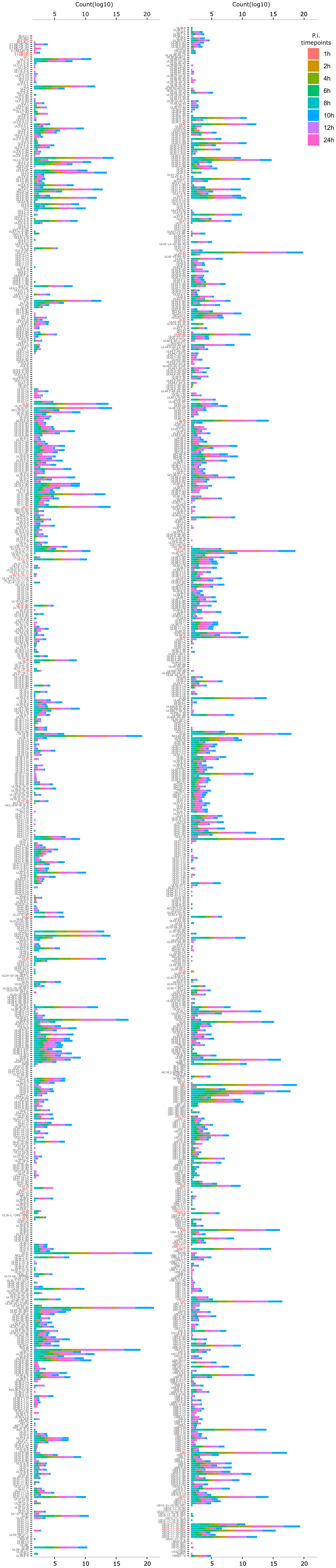

Supplement: Supplementary Figure 3 — The cumulative abundance of transcript isoforms. Transcript isoforms were annotated and counted in separate stages of the viral infection using the LoRTIA software suite. The names of isoforms annotated in previous works by other methods are in red, whereas the isoforms detected by long-read sequencing are in black. [file Image_3.tiff]
